# Effects of *N*-glycosylation on protein conformation and dynamics: Protein Data Bank analysis and molecular dynamics simulation study

**DOI:** 10.1038/srep08926

**Published:** 2015-03-09

**Authors:** Hui Sun Lee, Yifei Qi, Wonpil Im

**Affiliations:** 1 Department of Molecular Biosciences and Center for Computational Biology, The University of Kansas, 2030 Becker Drive, Lawrence, Kansas 66047, United States

## Abstract

*N*-linked glycosylation is one of the most important, chemically complex, and ubiquitous post-translational modifications in all eukaryotes. The *N*-glycans that are covalently linked to proteins are involved in numerous biological processes. There is considerable interest in developments of general approaches to predict the structural consequences of site-specific glycosylation and to understand how these effects can be exploited in protein design with advantageous properties. In this study, the impacts of *N*-glycans on protein structure and dynamics are systematically investigated using an integrated computational approach of the Protein Data Bank structure analysis and atomistic molecular dynamics simulations of glycosylated and deglycosylated proteins. Our study reveals that *N*-glycosylation does not induce significant changes in protein structure, but decreases protein dynamics, likely leading to an increase in protein stability. Overall, these results suggest not only a common role of glycosylation in proteins, but also a need for certain proteins to be properly glycosylated to gain their intrinsic dynamic properties.

Glycans are present in cells as covalent attachments to other molecules such as proteins (glycoproteins) or lipids (glycolipids), although isolated glycans can also bind to proteins as ligands^1^,^2^,^3^. These glycans have been linked to an increasing number of important biological processes^4^. The two main glycosidic linkages to proteins involve either nitrogen in the side chain of asparagine (*N*-linked glycans)^5^ or oxygen in the side chain of serine or threonine (*O*-linked glycans)^6^. Gangliosides containing sialic acid are a type of lipid glycosylation^7^. In addition, proteins can be attached to the membrane surface by a linkage between the carboxyl-terminal group and a glycophosphatidylinositol (GPI) anchor^8^.

Protein glycosylation is one of the most important post-translational modifications in the cell, and more than half of all proteins in nature are expected to be glycosylated^9^. *N*-linked glycosylation is a chemical process in which oligosaccharyltransferase catalyzes the *en* *bloc* transfer of the oligosaccharide portion of a lipid-linked oligosaccharide (LLO) onto the acceptor asparagine of nascent proteins, defined by the consensus sequon Asn-X-Thr/Ser (X ≠ Pro)^10^,^11^,^12^. The protein-linked glycan structure is then further processed and chemically derivatized. On the other hand, *O*-linked glycosylation begins with the addition of a single monosaccharide GalNAc (by a *N*-acetyl galactosaminyltransferase) to serine/threonine at a site which does not have a well defined sequence motif, and this GalNAc can be further elongated or modified^13^.

Protein modifications by *N*- or *O*-glycans modulate protein's biophysical properties and consequently regulate the function of the native protein encoded by the genome^14^. Numerous experiments have revealed that glycosylation can alter thermodynamic, kinetic, and structural features of proteins, conferring an additional information content beyond what is dictated by their sequence^15^. *N*-linked glycosylation is perhaps the most chemically complex and ubiquitous protein modification in all eukaryotes^11^. The large hydrophilic carbohydrates appended to proteins have been implicated in a myriad of biological processes, including modification on protein folding^16^, modulation of protein stability, oligomerization, and aggregation^15^,^17^, endoplasmic reticulum (ER) quality control and protein trafficking^18^, host cell-surface interactions^19^, and modulation of enzyme activity^20^.

There is considerable interest in developments of general approaches to predict the structural consequences of site-specific glycosylation and to understand how these effects can be exploited in protein design with advantageous properties. This knowledge is essential to develop glycoprotein therapeutics in modern medicine^21^. In this study, the impacts of *N*-glycans on the folded glycoproteins are investigated in terms of protein structure and dynamics in their glycosylated and deglycosylated forms using an integrated computational approach of the Protein Data Bank (PDB) structure analysis and atomistic molecular dynamics (MD) simulations. Our study reveals that *N*-glycosylation does not induce significant global/local changes in protein structure, but decreases protein dynamics, likely leading to an increase in protein stability.

## Results

### N-glycosylation does not significantly affect global and local protein structure

We first investigated the impact of *N*-glycosylation on proteins' global and local structures by measuring structural similarity between the glycosylated (GP) and deglycosylated (P) forms of identical proteins in the PDB (see Methods for detailed descriptions on the preparation of the PDB structure sets and the similarity measurement). The same analysis was also performed between deglycosylated forms (P/P pairs) for a comparison with the results from the GP/P pair.

**Figure 1:**
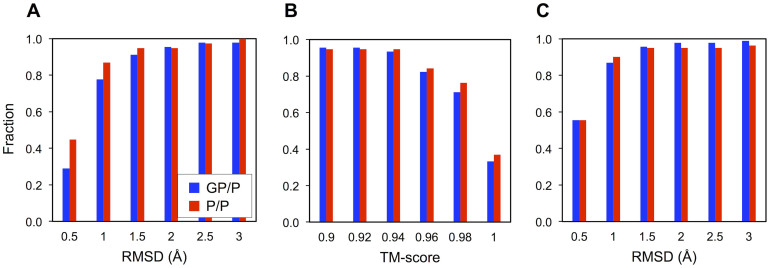
Global and local structural similarity measurement for PDB GP/P and P/P pair sets. GP and P stand for the glycosylated and deglycosylated forms of identical proteins, respectively. The global structural similarity is represented by the histograms of accumulated fractions in terms of (A) RMSD and (B) TM-score. (C) The local structural similarity is represented by a histogram of accumulated fractions in terms of local structure RMSD.

The global structural similarity is quantified by root-mean-square deviation (RMSD) and TM-score. The RMSD distribution in the GP/P set is comparable to that in the P/P set (Figure 1A). 91% of the GP/P pairs and 95% of the P/P pairs have the RMSD of ≤1.5 Å, i.e., most protein pairs do not show any significant conformational changes (Figure S1). We also performed visual inspection of all GP/P pairs whose RMSD is larger than 2.0 Å. Even in these cases, the conformational changes are not directly caused by glycosylation, but by other factors such as domain rotation (Figure S2A), the movement of flexible loops (Figure S2B), and folding into an intermediate state (Figure S2C). The RMSD is a quantity that is dependent on the protein size, i.e., its value is generally bigger as the protein length increases. To eliminate this size-dependency, a size-independent score, TM-score, is also used to measure the global structural similarity. As shown in Figure 1B, the distributions from the GP/P and the P/P sets become more analogous when the global structural similarity is evaluated by TM-score (93% of the GP/P pairs and 95% of the P/P pairs at TM-score of ≥0.94). These results indicate that in most cases, the global structures of glycosylated proteins are almost identical to deglycosylated ones, and thus the effect of *N*-glycosylation on the global protein structure is insignificant (at least in the protein structures currently available in the PDB). The impact of *N*-glycans on protein structure was also evaluated in terms of local structure RMSD using a radius of 15 Å (Figure 1C) from the Cα atom of the glycosylated Asn residue. Similar to the global structure comparison, no salient structural changes around the glycosylation sites are observed from most PDB glycoprotein structures.

**Figure 2:**
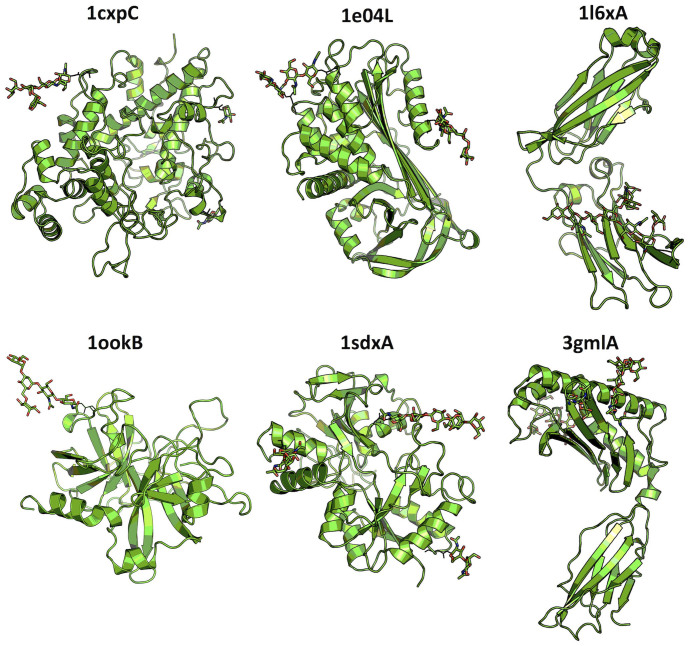
The cartoon representation of six glycoproteins (PDB id + chain id) used for MD simulation study. The stick representation is used to display *N*-linked glycans.

A systematic analysis of PDB protein structures can provide valuable insight into the extent of structural changes induced by *N*-glycosylation. However, the PDB glycoprotein structures are “static” and thus they cannot be directly used to extract the extent of changes in protein dynamics upon glycosylation. To better understand the effect of *N*-glycosylation on protein structure and dynamics, six representative glycoproteins were elaborately selected from the PDB, and three independent 200-ns MD simulations were performed for both glycosylated and deglycosylated forms of each protein (Tables S1 and S2; see Methods for detailed descriptions). The global topologies of the six representative glycoproteins are shown in Figure 2.

**Figure 3:**
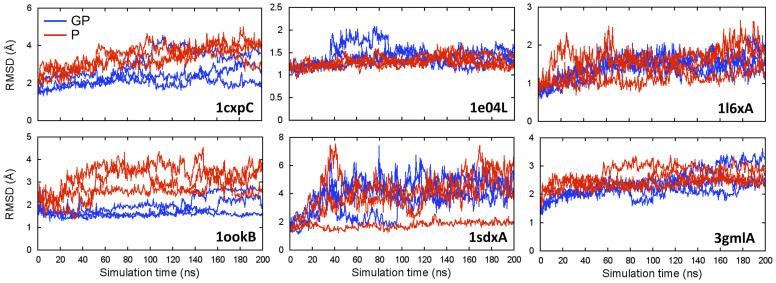
The RMSD time-series of glycosylated (GP, blue) and deglycosylated (P, red) proteins from 200-ns MD simulations. The RMSD was calculated with respect to the initial structure. Each line represents an independent run.

**Figure 4:**
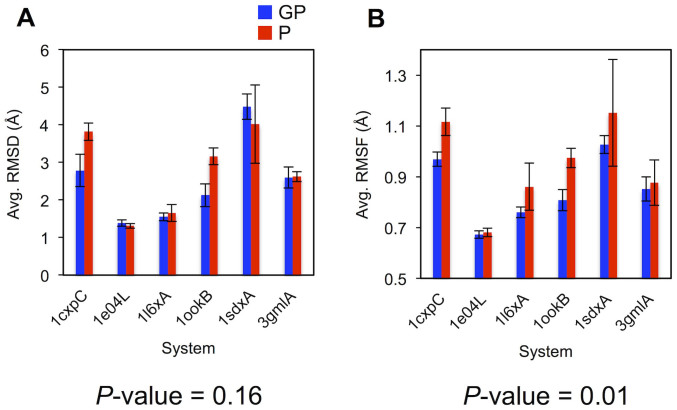
Histograms showing the RMSD and RMSF differences between glycosylated (GP, blue) and deglycosylated (P, red) proteins during the last 50-ns MD simulations. (A) The average RMSDs calculated using the last 50-ns trajectories (from Figure 3). (B) The average RMSFs over all protein residues (from Figure 5). In the plots, each value is the mean of the average RMSDs or RMSFs from the three independent runs, and the error bars represent the standard errors. *P*-values were calculated using a paired *t*-test.

The RMSD with respect to the initial structure is plotted as a function of simulation time in Figure 3 for each glycoprotein system. Figure 4A shows the means and standard errors of the average RMSDs from the last 50 ns for the three replicates. A comparison of the mean RMSDs between glycosylated and deglycosylated proteins indicates that glycosylation does not significantly affect proteins' global structures (*P*-value = 0.16), which is in agreement with the results from the PDB structure analysis. 1cxpC is the system showing the largest mean RMSD difference between the glycosylated and deglycosylated protein, but the magnitude is still around 1 Å.

### N-glycosylation decreases protein dynamics

Our PDB analysis and MD simulations demonstrate that *N*-glycosylation does not cause large conformational changes in proteins. To further investigate the effect of *N*-glycosylation on protein dynamics, the fluctuations of each residue are analyzed by root-mean-square fluctuations (RMSFs) (see Methods for detailed descriptions).

**Figure 5:**
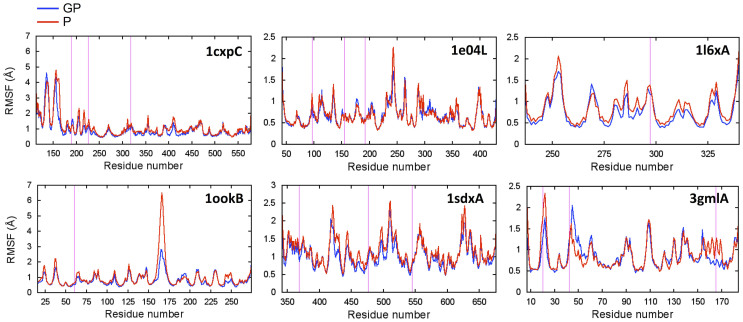
The RMSF plots of glycosylated (GP, blue) and deglycosylated (P, red) proteins calculated from the last 50-ns trajectories. The average structure of the last 50 ns was used as the reference structure to calculate the RMSFs. The magenta lines in each plot correspond to the glycosylation sites. The plots are the averages of the three independent replicates, and the errors bars are not displayed for clarity.

The per-residue RMSF plots (Figure 5) and the mean RMSF histogram (Figure 4B) clearly show that all deglycosylated proteins in the benchmark systems are more dynamic than their glycosylated forms with statistical significance (*P*-value < 0.05). The RMSF is increased by deglycosylation at most of the glycosylation sites in the benchmark systems (11 out of 14 glycosylation sites in Table S3). Interestingly, as shown in the RMSF plots (Figure 5), the residues showing the largest RMSF decrease upon glycosylation do not necessarily correspond to the residues near the glycosylated sites, implying that the impact of glycosylation is not localized but can be propagated to other regions of the protein. In system 1ookB, for example, the glycan is attached at Asn-60, whereas the residue (Pro-166) showing the largest RMSF difference upon glycosylation is located far from the glycosylation site; the Cα atom distance between the two residues is 33.7 Å (Figure S3). This allosteric change is a frequently observed phenomenon in protein dynamics. It is known that a perturbation caused by a change of molecular environment is propagated to other distant regions of the protein to redistribute the protein's dynamics in order to minimize the potential entropy loss due to the modification^22^,^23^.

## Discussion

In this study, we have investigated the effects of *N*-glycosylation on protein structure and dynamics using experimentally solved three-dimensional structures deposited in the PDB and atomistic MD simulations. Recently, Xin and Radivojac collected a set of PDB glycoprotein clusters and then compared the average RMSD from their GP/P pairs with that from their P/P pairs for each cluster^24^. Although their approach is similar to ours in that they also used the PDB structures for the analysis, the detailed methods of structure set preparation and data analysis are largely different. They mentioned that glycosylation induces significant (yet not extreme) conformational changes at both local and global level. However, our results from the PDB structure analysis and MD simulations indicate that *N*-glycosylation does not induce significant conformational changes in folded protein structures.

Our RMSF analysis from the MD trajectories of six representative glycoproteins and their deglycosylated forms shows that *N*-glycosylation decreases the dynamic fluctuation of the protein. The impact of glycosylation is not localized at the glycosylation site, but can be propagated to other regions of the protein. Experimental evidences are in good agreement with our results. For example, carbon-13 NMR spectroscopic studies of native and sequentially deglycosylated ovine submaxillary mucin show that the Cα atoms of the glycosylated Ser and Thr residues are considerably more constrained than their deglycosylated counterparts. A Gly residue located next to the glycosylated Ser also exhibits increase in motion upon removal of GalNAc, indicating that the effects of glycosylation extend to residues beyond the amino acids directly bound to carbohydrate^25^. NMR measurement of amide-proton/deuterium exchange rates shows that glycosylation of ribonuclease B leads to the protection of amide-proton resonances from solvent exchange for a large number of residues, both in the vicinity of and away from the glycosylation site, compared to the deglycosylated form. This result indicates that the presence of a sugar enhances the protein stability^26^. The proteinase inhibitor PMP-C was examined to investigate the effects of threonine-linked L-fucose moiety on the structure, dynamics, and stability of the protein by NMR spectroscopy. The overall backbone conformations of fucosylated and defucosylated PMP-C are very similar and cannot be distinguished from one another. The linking of an L-fucose moiety to PMP-C has only a local structural effect, predominantly on the side chains of neighboring residues. A comparative analysis of the exchange rates of amide protons indicates that fucosylation is responsible for an overall decrease in the dynamic fluctuations of the molecules, leading to an increase in stability as examined by thermal denaturation^27^.

We have performed additional 200-ns MD simulations for both glycosylated and deglycosylated forms of ribonuclease B, which is an experimentally validated representative glycoprotein, aiming at comparing the simulation results with the experimental observables. A decreased RMSF (0.91 ± 0.04 Å for glycoprotein and 1.03 ± 0.08 Å for deglycosylated protein) is observed upon glycosylation (Figure S4). There is a correlation of lower structural fluctuations detected by MD simulations with the increased stability of the protein upon glycosylation by experimental measurements. Therefore, the MD simulation techniques can be used to computationally investigate the effects of glycosylation on the structural stability of target proteins. However, it would be interesting to see if slight difference in terms of RMSF, observed from some benchmark targets (e.g., 1e04L and 3gmlA in Figure 4B), can indeed lead to detectable difference in their stability upon glycosylation.

The molecular mechanisms underlying the decreased dynamics upon glycosylation could be explained in several ways. *N*-glycans could act like molecular glue, holding the residues around the glycosylation sites together through the favorable interactions, resulting in stabilizing the protein structures. We have characterized hydrogen bonds in the crystal structures (Figure S5) and measured van der Waals and electrostatic energy during the MD simulations (Table S4). Favorable interactions between the protein and the glycan components are observed in all the benchmark *N*-glycoproteins. It is also known that increasing glycan molar contents correlates with the decreased structure dynamics of the protein^28^. However, we could not find a clear correlation of the magnitude of the decreased dynamics with either favorable interactions or glycan molar mass (Table S3), suggesting the importance of glycosylation sites and protein geometry.

A potential limitation of the PDB glycoprotein analysis is that the current PDB glycoprotein library is not complete but biased toward specific protein families that are experimentally easy to handle or scientifically more interesting^29^. Although glycosylation of a protein generally increases its thermodynamic stability compared to that of deglycosylated protein, it has also been reported that glycosylation reduces the thermodynamic stability of tyrosinase and tyrosinase-related proteins^30^,^31^. Therefore, as our PDB dataset is limited, exceptions to our general conclusion can also be found.

More than half of all proteins in nature are expected to be glycosylated, but only a small portion of X-ray crystallographic structures in the PDB (~4% as of March 2013) includes covalently linked *N*- or *O*-glycans. This small number of glycoproteins in the PDB is mainly because most of the target proteins are partially or fully deglycosylated prior to crystallization to remove the glycans that prevent or reduce favorable crystal contacts^32^. Other reasons could be micro-heterogeneity of glycans, the inherent flexibility of glycans, and the use of non-native protein expression systems. In addition to scientific insight into the effects of glycosylation on protein structure and dynamics, our study also proposes that accounting for proper glycosylation may be needed to more reliably address dynamic properties of PDB X-ray crystallographic structures in that glycosylation may affect the dynamic properties of target proteins.

## Methods

### Preparation of glycosylated/deglycosylated (GP/P) and deglycosylated/deglycosylated (P/P) protein pair sets

**Figure 6:**
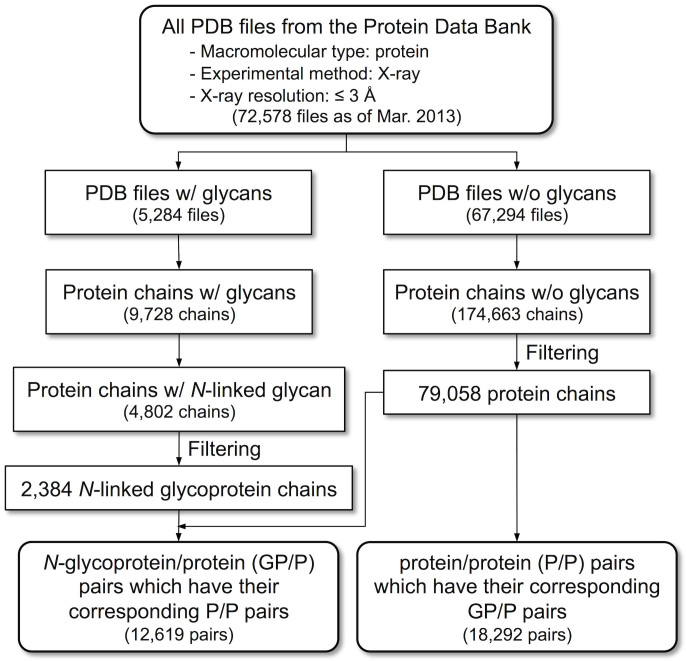
The schematic illustration of the overall procedure used to prepare the glycoprotein structure pair sets (GP/P and P/P) from the PDB.

Figure 6 summarizes the overall procedure to prepare glycoprotein structure pairs for PDB structure analysis. We downloaded the PDB files of X-ray crystallographic structures that contain at least one protein and their resolution is ≤3 Å (72,578 files as of March 2013). For automatic sugar identification in the PDB files, we used *Glycan Reader*^33^. *Glycan Reader* detected 5,248 carbohydrate-containing PDB files (~7%) among all the downloaded files. A total of 9,728 protein chains that include covalently linked glycans or interact with glycan ligands were extracted. The protein chains with the covalently attached glycans were subsequently divided into *N*-linked and *O*-linked glycoproteins. A total of 4,802 protein chains were designated to *N*-linked glycoprotein chains (i.e., *N*-glycoproteins). The *N*-glycoproteins were then filtered to remove redundancy in each PDB file with a 90% sequence identity cutoff. The *N*-glycoproteins with less than 50 amino acids were also discarded because short proteins often do not have well-defined tertiary structures. A total of 2,384 *N*-glycoproteins were retained after the filtering process. Protein chains were also individually extracted from the downloaded PDB files that do not contain any carbohydrate molecules, followed by the same filtering process to remove redundant protein chains and small proteins. Finally, there were a total of 79,058 protein chains without associated carbohydrates.

To prepare a list of pairs between *N*-glycosylated proteins and the same, but deglycosylated proteins in the PDB, the identical sequence of each *N*-glycoprotein chain (i.e., 100% sequence identity) was searched against the sequences of the deglycosylated protein chains using the stand-alone BLAST (ftp://ftp.ncbi.nlm.nih.gov/blast/executables/LATEST-BLAST/). Hereafter, we refer to this protein pairs as the GP/P set. The number of pairs in this set was 12,619, which is bigger than the number of *N*-glycoproteins in the final data set (2,384) because there are multiple glycosylated and deglycosylated protein structures (from different PDB files) and we include them to consider the effect of differences in X-ray crystallographic conditions. Similarly, we also prepared a list of protein pairs that do not have any carbohydrate molecules. This set is called the P/P (deglycosylated/deglycosylated) set. In this case, the sequences of deglycosylated proteins in the GP/P set were used as query for the BLAST search against the sequences of protein chains that do not contain carbohydrate molecules, aiming at using only P/P pairs whose proteins belong to the GP/P set. There were a total of 18,292 P/P pairs.

### Measurement of structural similarity in GP/P and P/P sets from the PDB structures

To measure the global structural similarities, each protein structure pair in both GP/P and P/P sets were superposed using a structure alignment tool, TM-align^34^. The global structure similarity was then evaluated by two quantities: root-mean-square deviation (RMSD) and template modeling score (TM-score). The TM-score is a protein size-independent quantity to measure the global structural similarity between two proteins ranging between 0 and 1, where 1 indicates a perfect match between two structures. Only Cα atoms were used for RMSD and TM-score calculations. The structure pairs in the GP/P and P/P sets were clustered using 100% sequence identity and an average over all pairs in each cluster was used to produce cumulative histograms as a function of RMSD and TM-score.

The Cα-RMSD around an *N*-glycosylation site was also measured for the GP/P and P/P sets. These local RMSDs were calculated separately for every glycosylation site in a protein; *N*-glycoprotein local structures were defined as concentric shells by a radius of 15 Å from the Cα atom of a glycosylated Asn residue. The glycosylated residues in each *N*-glycoprotein were obtained from *Glycan Reader*. We identified the equivalent residues in the corresponding deglycosylated protein by performing a global sequence alignment between the sequences of glycosylated and deglycosylated proteins; although two protein chains have 100% sequence identity, calculating their local RMSD using equivalent residue pairs is often tricky due to mismatches in the sequence length and residue number. Local structure pairs were superposed by a least squares fitting of the equivalent residue pairs to calculate the RMSD. Similar to the global structure comparison, the GP/P and P/P pairs were clustered in terms of 100% sequence identity and glycosylation site, and the average RMSDs for each cluster were used for the histogram analysis.

### Molecular dynamics simulations

In order to select the representative *N*-glycoproteins for MD simulations, all the PDB *N*-glycoprotein chains of identical sequences were first clustered and then the protein chains with the largest number of PDB files were chosen from each cluster. The protein chains with less than 10 PDB files were discarded from the cluster list. The number of residues in each glycan was counted for each *N*-glycoprotein. Six protein chains were finally selected as the representative *N*-glycoprotein systems based on the number of the PDB files, the maximum number of glycan residues, the number of amino acid residues, and the simulation system size (Tables S1 and S2). For preparation of deglycosylated proteins, carbohydrate molecules in the six glycoproteins were deleted from the structure files. Seven missing residues (196–202) in the crystal structure of system 3gmlA were modeled using a loop modeling method, ModLoop^35^.

To simulate the glycoproteins and deglycosylated forms, we have followed the general procedure of system building and equilibration in *Quick MD Simulator* integrated with *Glycan Reader*^33^ in CHARMM-GUI (http://www.charmm-gui.org)^36^. All the molecules except corresponding *N*-glycosylated protein, glycans, and structurally important ions were removed. The N- and C-termini were capped with acetyl (ACE) and N-methyl (CT3) groups, respectively. The TIP3P model was used for explicit water molecules. The cubic system size was determined to have at least 10 Å from the protein in each XYZ direction, and 150 mM KCl was added. The system information is given in Table S2.

The CHARMM36 force field^37^,^38^,^39^,^40^,^41^ was used for the proteins and carbohydrates, respectively. All calculations were performed at 300 K. The particle mesh Ewald algorithm^42^ was applied to calculate electrostatic forces, and the van der Waals interactions were smoothly switched of at 10–12 Å by a force-switching function^43^. A time step of 2 fs was used in all simulations. After short constant particle number, volume, and temperature (NVT) equilibration using CHARMM^44^, NAMD^45^ was used for 5-ns constant particle number, pressure, and temperature (NPT) equilibration with restraints and additional 5-ns equilibration without restraints for each system. To assure gradual equilibration of the system, positional restraints for backbone and side chain heavy atoms were applied and the restraint forces were gradually reduced during the equilibration. Additional dihedral angle restraints were applied to restrain all the sugar rings to the pertinent chair conformation. For NAMD NPT simulations, Langevin coupling coefficient was set to 1 ps^−1^ and a Nosé-Hoover Langevin-piston^46^,^47^ was used to maintain constant pressure (1 bar) with a piston period of 50 fs and a piston decay of 25 fs.

Each system was further simulated for 200 ns on Anton^48^ using the CHARMM36 force field. The NVT ensemble was used with the temperature maintained at 300 K using the Nosé-Hoover method. The time step was 2 fs and trajectories were saved every 240 ps. The short-range forces and long-range electrostatics were evaluated every 2 fs and 6 fs, respectively. The short-range nonbonded and electrostatic interactions were calculated with a cutoff of 9.52 Å. The long-range electrostatic interactions were calculated using the k-Gaussian Split Ewald method^49^ with a 64 × 64 × 64 grid. SHAKE was used to constrain all bonds involving hydrogen atoms. Three independent MD simulations were performed for both glycosylated and deglycosylated forms of each system.

### Analysis of MD Simulation Trajectories

Two quantities were measured from the MD simulation trajectories and compared between glycosylated and deglycosylated proteins: global RMSD and root-mean-square fluctuation (RMSF) for each residue. Only Cα atoms were used for these calculations. The RMSD was calculated with respect to the initial PDB structure for each simulation trajectory. For the RMSF calculation, the average structure of the last 50 ns was used as the reference structure. To quantitatively compare dynamic properties between glycosylated and deglycosylated proteins, the mean RMSDs and RMSFs with the standard errors during the last 50 ns were calculated over the three independent replicates.

We have performed one-tailed paired *t*-test to compare the mean RMSD (or RMSF) with the null hypothesis that the RMSDs (or RMSFs) of glycosylated and deglycosylated proteins are identical and the alternative hypothesis that the RMSD (or RMSF) of deglycosylated proteins is larger than that of glycosylated ones. The hypothesis tests for the average RMSD and RMSF were evaluated with the significance level of 0.05.

1l6xA and 3gmlA systems (Figure 2) showed abnormally large conformational changes during the simulations due to their unique topology that consists two domains, but the two domains do not have strong interactions with each other (Figure S6). Since we are interested in the impact of glycans on protein structure and dynamics, we used only domains containing glycans (residues 237–340 for 1l6xA and 7–184 for 3gmlA) for the trajectory analysis.

## Author Contributions

H.S.L. and W.I. designed the study and experiments. H.S.L., Y.Q. and W.I. prepared manuscript. H.S.L. and Y.Q. performed the experiments.

## Supplementary Material

Supplementary Information Supplementary info
