# Supplementary material for: Effects of N-glycosylation on protein conformation and dynamics: Protein Data Bank analysis and molecular dynamics simulation study
Source: Sci Rep. 2015 Mar 9;5:8926. doi: 10.1038/srep08926 (PMC4352867; doi:10.1038/srep08926)

Supplementary Information

Effects of *N*-glycosylation on protein conformation and dynamics: protein data bank analysis and molecular dynamics simulation study

Hui Sun Lee, Yifei Qi, and Wonpil Im

Department of Molecular Biosciences and Center for Computational Biology, The University of Kansas, 2030 Becker Drive Lawrence, Kansas 66047, United States

Table S1. The most redundant glycoproteins in the PDB and six representative proteins (bold) chosen for MD simulation study. The last letter in the PDB names corresponds to the protein chain ID.

Cluster	Protein name	# of PDB files	Max. # of sugar residues	PDB of max. # sugars	# protein residues
**1**	**α-Thrombin**	**63**	**5**	**1ookB**	**232**
2	Plant Acetylcholinesterase	50	2	1dx6A	532
3	α-mannosidase II	36	1	1htyA	1014
**4**	**Lactoferrin**	**36**	**6**	**1sdxA**	**335**
**5**	**CD1D antigen**	**34**	**6**	**3gmlA**	**275**
6	Renin	31	2	3gw5A	337
7	Lactoperoxidase	29	3	2gj1A	580
8	Human dipeptidyl peptidase IV (1)	22	4	2ripA	729
9	Mouse Acetylcholinesterase	22	2	1j06A	542
10	Ribosome inactivating protein	22	2	3mrwA	246
11	Glutamate carboxypeptidase	21	4	2c6cA	694
12	Acid-β-glucosidase	19	5	2j25B	497
13	Integrin β-3	19	7	2vdkB	461
14	ANCE	16	6	2x8yA	595
15	α-amylase	16	1	1bsiA	496
16	Human Dipeptidyl peptidase IV (2)	15	2	1x70B	728
17	Influenze virus hemagglutinin	14	2	2ypgB	173
**18**	**Immunoglobulin γ-1 heavy chain**	**11**	**10**	**1l6xA**	**207**
19	Butyryl cholinesterase	11	3	1p0iA	526
20	H-2 class I histocompatibility antigen, K-B α chain	10	3	1g7pA	274
**21**	**Myeoloperoxidase isoform C**	**10**	**6**	**1cxpC**	**466**
22	Maltase-glucoamylase	10	2	2qlyA	568
23	Integrin α-IIB	10	2	3fcsC	959
**24**	**Antithrombin**	**10**	**5**	**1e04L**	**389**
25	Bovine rhodopsin	10	4	1hzxB	326

Table S2. MD simulation system information.

System name	Glycan	# water	# ions	# total atoms
1cxpC	w/	25,234	63 K^+, 80 Cl^-	83,512
	w/o	25,292	63 K^+, 80 Cl^-	83,495
1l6xA	w/	23,116	67 K^+, 67 Cl^-	73,012
	w/o	23,165	67 K^+, 67 Cl^-	72,926
1e04L	w/	23,754	70 K^+, 66 Cl^-	77,865
	w/o	23,835	70 K^+, 66 Cl^-	77,883
1ookB	w/	13,940	37 K^+, 42 Cl^-	46,187
	w/o	11,737	31 K^+, 36 Cl^-	39,449
1sdxA	w/	17,962	51 K^+, 52 Cl^-	59,316
	w/o	18,854	54 K^+, 55 Cl^-	61,731
3gmlA	w/	26,190	77 K^+, 73 Cl^-	83,321
	w/o	26,318	77 K^+, 73 Cl^-	83,424

Table S3. Glycosylation site information and RMSF differences at the glycosylation site. $\Delta RMSF_i = RMSF_{i,P} - RMSF_{i,GP}$, where i is for the glycosylated site, GP for glycosylated protein, and P for deglycosylated protein. The average ΔRMSF is the average of $\Delta RMSF_i$ over all the residues in a protein. $\Delta RMSF_i$ is the average of the three independent replicates. The standard errors are omitted for clarity.

PDB	# of glycans	Glycosylation site	# of sugar residues	Location of glycosylation site	$\Delta RMSF_i$	Avg. ΔRMSF
		Asn-189	1	Loop	0.05	
1cxpC	3	Asn-225	1	Loop	0.13	0.15
		Asn-317	6	Loop	0.16	
		Asn-96	2	Loop	0.34	
1e04L	3	Asn-155	2	Loop	−0.01	0.01
		Asn-192	5	α-helix	−0.02	
1l6xA	1	Asn-297	10	Loop	0.22	0.10
1ookB	1	Asn-60	5	Loop	0.08	0.17
		Asn-368	2	Loop	0.26	
1sdxA	3	Asn-476	3	α-helix	0.21	0.12
		Asn-545	6	α-helix terminal	0.08	
		Asn-20	1	β-sheet terminal	0.44	
3gmlA	3	Asn-42	5	Loop	−0.22	0.02
		Asn-165	6	α-helix	0.50	

Table S4. Average van der Waals (E_{vdW}) and electrostatic (E_{elec}) energies between the protein and the glycans during the last 50-ns simulations. The average is over the three independent replicates with the standard errors. These values should be considered carefully as E_{vdW} becomes negatively larger when the number of sugar units in glycans increases as well as E_{elec} is given without consideration of electrostatic solvation contributions. Nonetheless, these numbers are provided here to illustrate that there are favorable interactions between the protein and the glycan components.

System	E_{vdW}	E_{elec}	$E_{vdW} + E_{elec}$
1cxpC	−33.38 ± 0.67	−89.69 ± 8.78	−123.17 ± 9.15
1e04L	−21.30 ± 0.42	−87.26 ± 1.67	−108.56 ± 1.24
1l6xA	−35.86 ± 7.21	−103.22 ± 14.20	−139.08 ± 20.16
1ookB	−7.67 ± 1.23	−41.86 ± 4.98	−49.53 ± 5.79
1sdxA	−42.92 ± 1.85	−143.22 ± 7.86	−186.14 ± 9.37
3gmlA	−38.71 ± 2.26	−96.73 ± 2.08	−135.44 ± 2.78

Figure S1. Two representative examples of the GP/P pairs having an RMSD of ~1.5 Å: (A) H-2 class I histocompatibility antigen, K-B α chain and (B) Triacylglycerol lipase. The glycosylated and deglycosylated proteins are colored in green and blue, respectively.

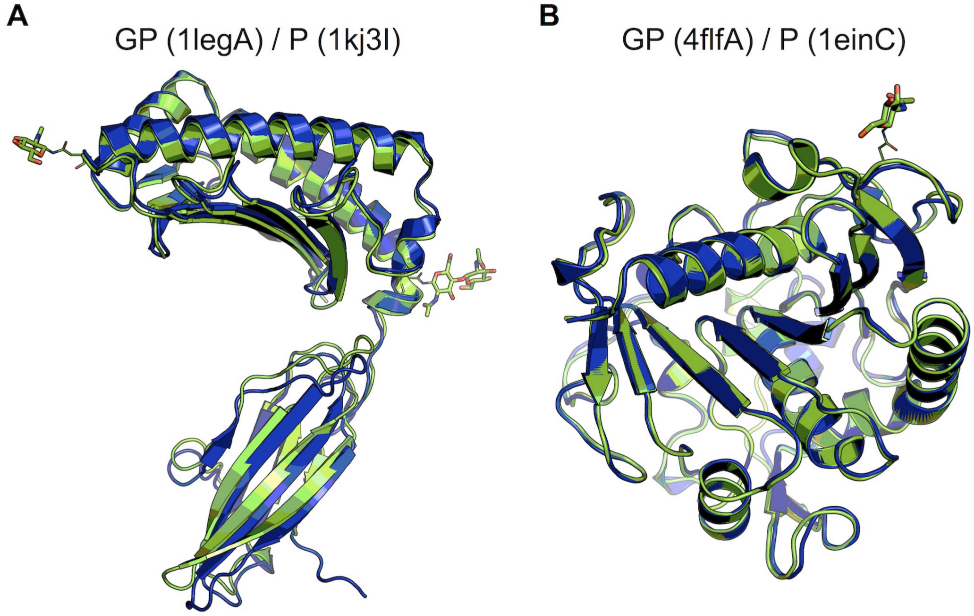

Figure S2. Three examples of the GP/P pairs having an RMSD of > 2.0 Å. (A) Human serum transferrin: GP (3qytA) / P (3v83A). The RMSD of the whole structures is 3.96 Å, but the RMSD becomes 0.54 Å only with the glycan-containing C-terminal domains. (B) SARS-coronavirus spike protein: GP (2ajfE) / P (2ghvC). (C) Human α1-antitrypsin: GP (7apiA) / P (2qugA). In all figures, the glycosylated and deglycosylated proteins are colored in green and blue, respectively.

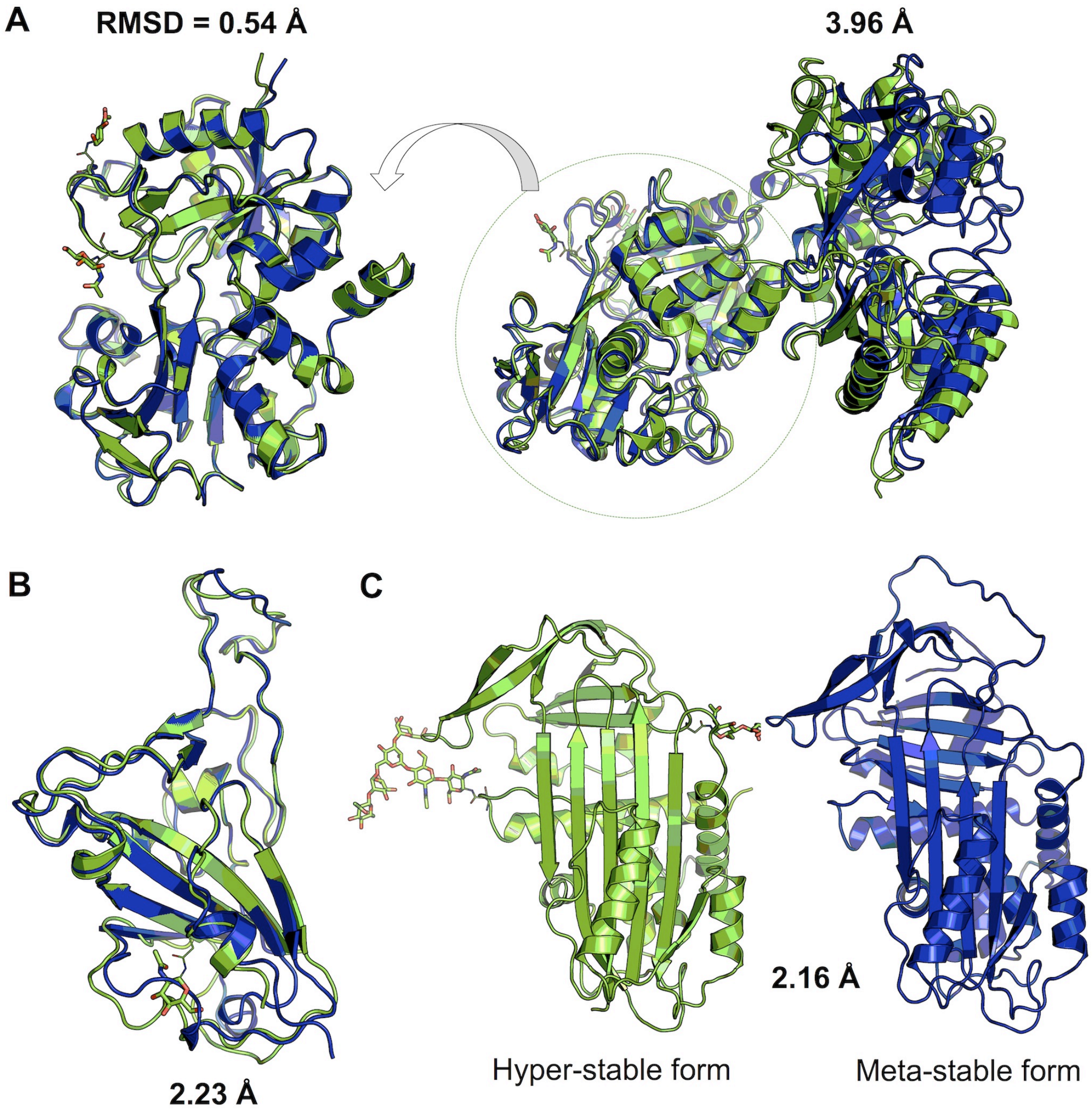

Figure S3. The crystal structure of 1ookB and the RMSF plots of the glycosylated (GP, blue) and deglycosylated (P, red) forms. In this *N*-glycoprotein, the glycan is attached at Asn-60. The three residues showing the largest RMSF differences upon glycosylation (residues 165−167) are colored in red in the glycoprotein structure.

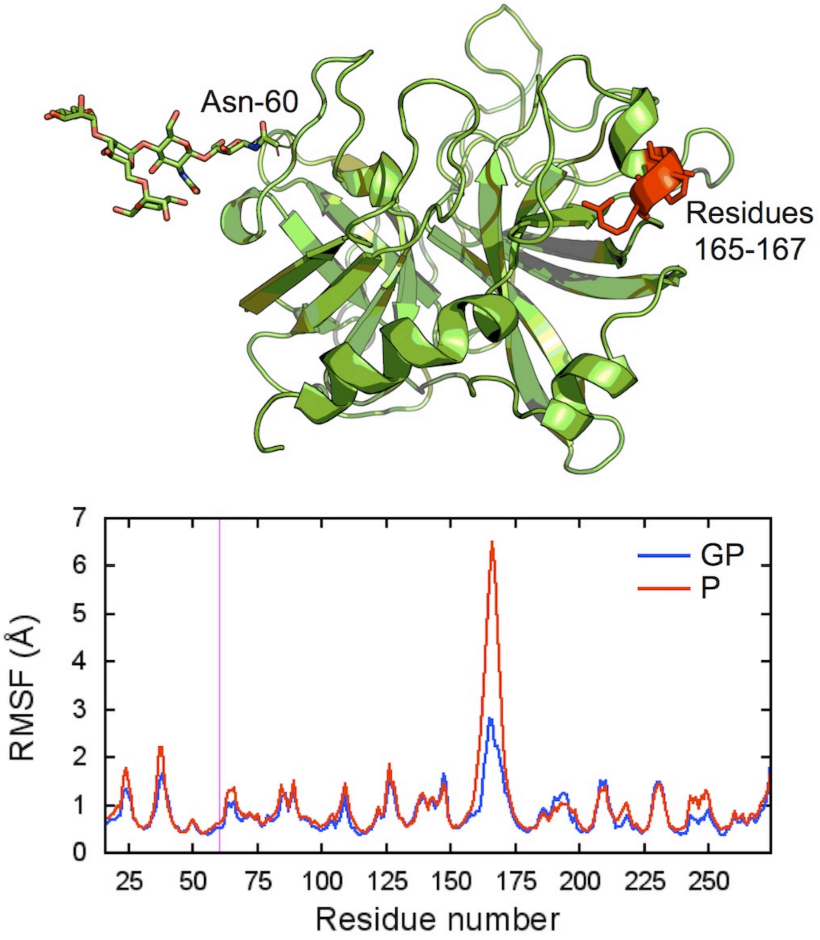

Figure S4. The crystal structure of ribonuclease B (1rbbA) and the RMSF plots of glycosylated (GP, blue) and deglycosylated (P, red) forms from the last 50-ns trajectories. The profile is the average of three independent replicates. The errors bars are not displayed for clarity. The magenta line in the plot corresponds to the glycosylation site.

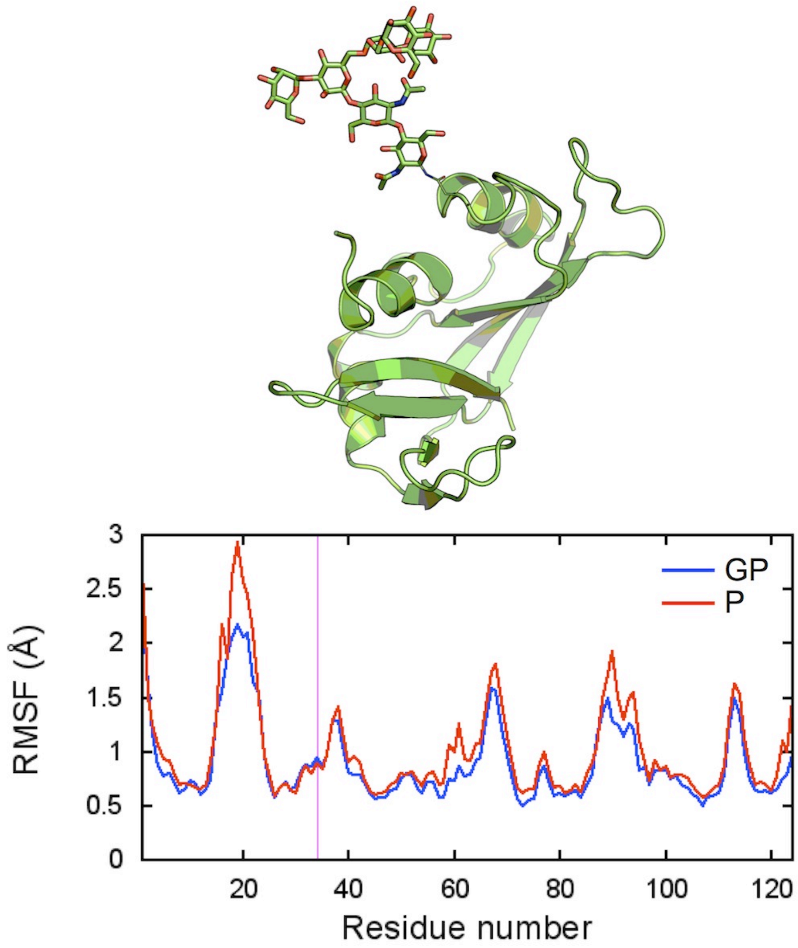

Figure S5. Characterization of hydrogen bonds (HBs) between protein and glycan components in the crystal structures of benchmark *N*-glycoproteins for MD simulations. Four out of six proteins have protein-glycan HBs.

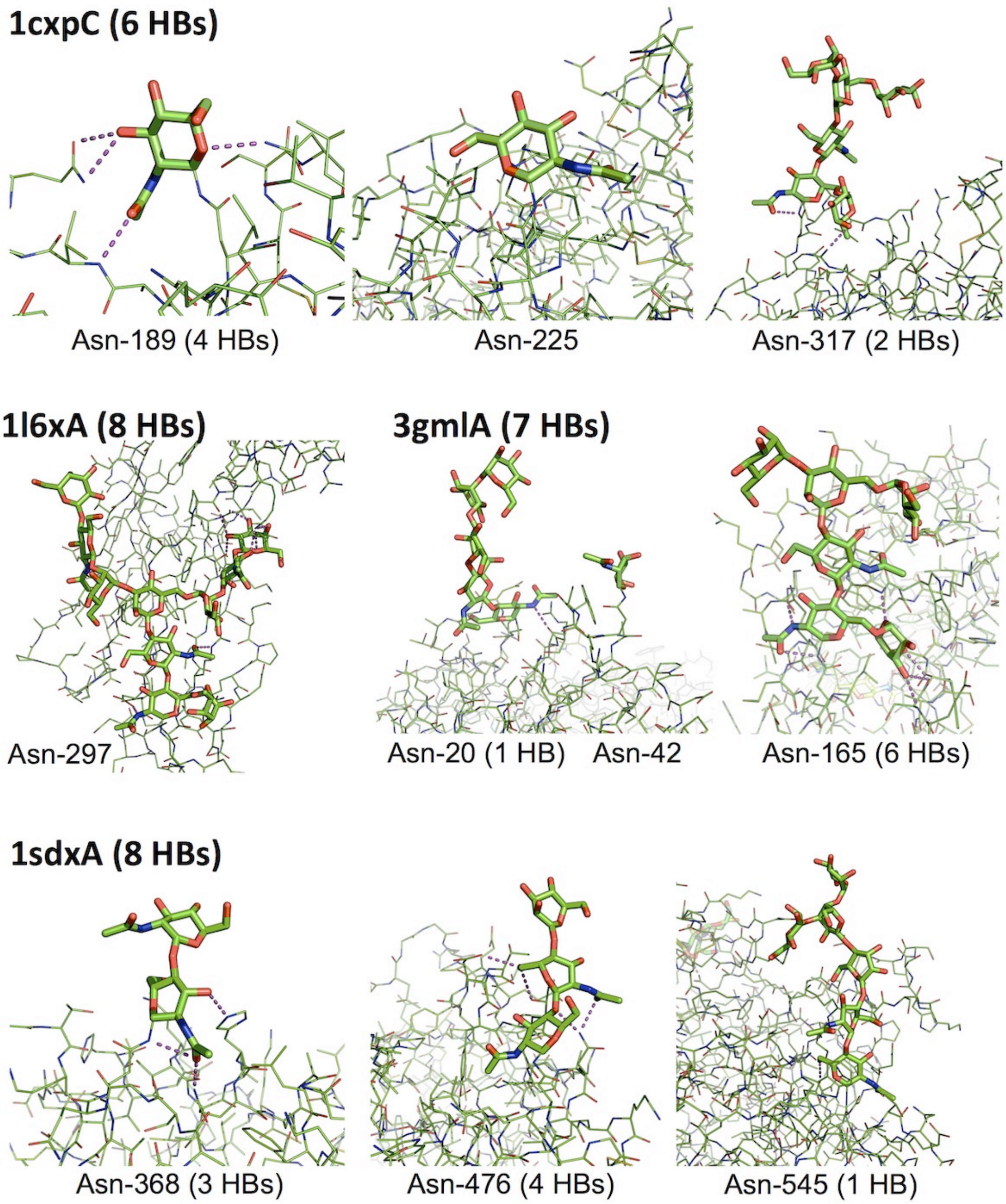

Figure S6. Large conformational changes in 3gmlA during MD simulations. The RMSD time-series calculated for (A) the whole protein and (B) a domain containing glycans (residues 7−184). In the plots, GP (blue) and P (red) represent the glycosylated and deglycosylated forms of the protein, respectively. (C) Structural comparison of the initial structure with a snapshot at 100 ns.

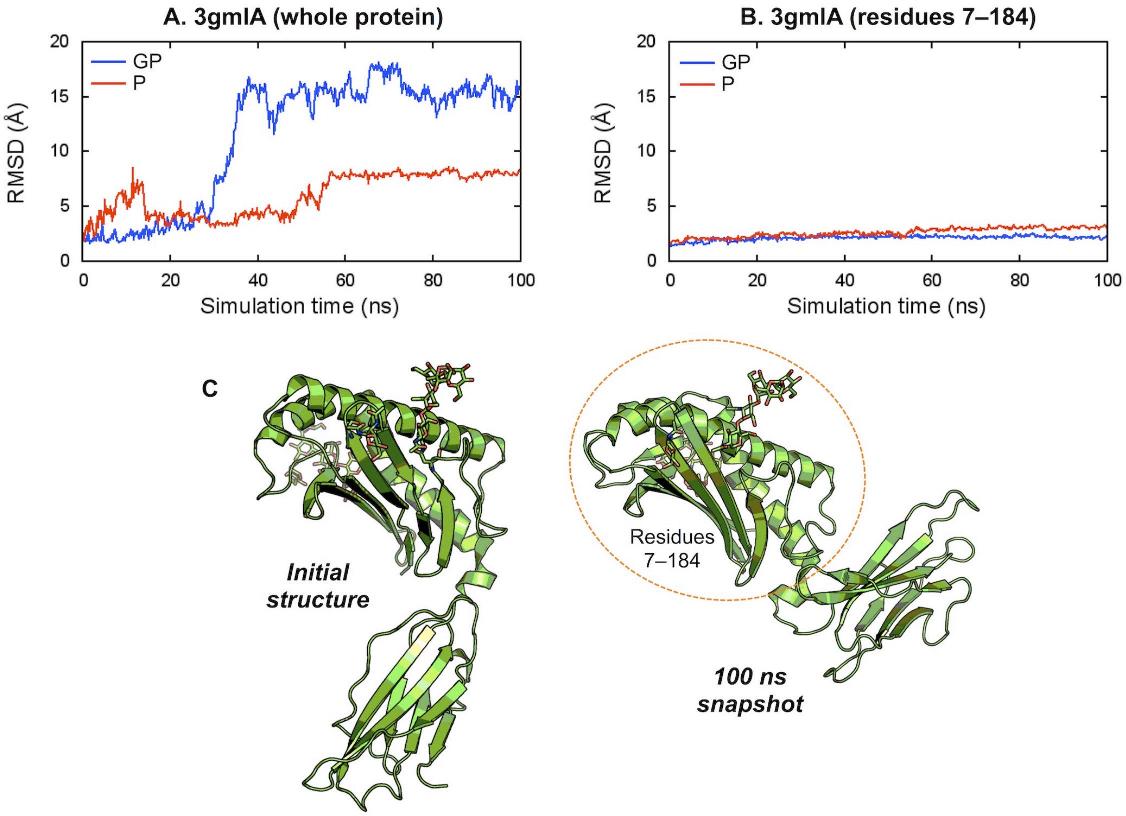

Supplement: Supplementary Information — Supplementary info [file srep08926-s1.pdf]
